# Editorial: Research reproducibility and preventing fraud

**DOI:** 10.3389/fcvm.2022.979467

**Published:** 2022-08-01

**Authors:** Shizuka Uchida, Jun Yu, Michel Goldman, Reto Asmis, Xiaofeng Yang, Elena Aikawa, Christos Bourantas, Masanori Aikawa, Hendrik Tevaearai Stahel

**Affiliations:** ^1^Center for RNA Medicine, Department of Clinical Medicine, Aalborg University, Copenhagen, Denmark; ^2^Department of Cardiovascular Sciences and Center for Metabolic Disease Research, Lewis Katz School of Medicine, Temple University, Philadelphia, PA, United States; ^3^Institute for Interdisciplinary Innovation in Healthcare, Université Libre de Bruxelles, Brussels, Belgium; ^4^Department of Internal Medicine, Wake Forest School of Medicine, Winston-Salem, NC, United States; ^5^Department of Cardiovascular Sciences, Centers for Metabolic Disease Research, Temple University Lewis Katz School of Medicine, Philadelphia, PA, United States; ^6^Division of Cardiovascular Medicine, Department of Medicine, Center for Interdisciplinary Cardiovascular Sciences and Center for Excellence in Vascular Biology, Brigham and Women's Hospital and Harvard Medical School, Boston, MA, United States; ^7^Institute of Cardiovascular Science, University College London, London, United Kingdom; ^8^Department of Cardiology, Barts Heart Centre, Barts Health NHS Trust, London, United Kingdom; ^9^Centre for Cardiovascular Medicine and Devices, William Harvey Research Institute, Queen Mary University of London, London, United Kingdom; ^10^Bern University Hospital, University of Bern, Bern, Switzerland

**Keywords:** data reproducibility, fraud prevention, journals, predatory journals, statistics

## Introduction

The reproducibility of experimental data is an essential part of scientific research as it is the absolute requirement that the results presented in a scientific manuscript must be reproduced by other studies. If such validation fails, which happens rather frequently (1–3), the most integral part of science is violated, which results in inconsistent scientific data presented to the public. The damages might be considerable since reproducibility issues lead to overall mistrust in science, pave the way for complotism and jeopardize academia-industry partnerships, which are essential to translate scientific advances into innovation. One of the devastating consequences is that often there is reviewers' bias who often reject studies with different findings from the first published study in the field resulting in a loss of people's efforts, money, time, and more importantly of useful information that could guide further research in the field. Many funding bodies have thus set strict rules for preventing research fraud resulting from intentional data manipulations. Considering the increasingly complex nature of modern scientific research with many different experimental techniques or study protocols employed to conduct studies, Frontiers in Cardiovascular Medicine introduced this Research Topic to provide a set of guidelines to increase research reproducibility and prevent fraud.

## Challenges and solutions for authors and editors

As the research methodologies become more complex with many different techniques employed to test a scientific hypothesis from various research angles, unintentional errors or misinterpretation of the experimental or clinical results can occur, which should be identified by the peer review process of journals. Thus, it is of utmost importance that reviewers are experts in the field and have a deep understanding of the statistical methods so as to evaluate the validity of the results and the conclusions drawn by the authors presenting the data. Not all the reviewers are familiar with all the experimental techniques and thus it is essential for a manuscript to be reviewed by several researchers with different and complementary scientific backgrounds. The same strategy applies to statistical methods, which has resulted in the appointment of expert statisticians—technical editors—to evaluate the validity of statistical methods, as well as the registration of clinical trials to ensure high ethical standards. As the number of journals continues to increase and so does the number of submitted manuscripts, securing several expert reviewers for each manuscript has become challenging, which overwhelms many handling editors. Aside from the required duties of journals to prevent research fraud, different public evaluation systems, such as PubPeer, are in place to monitor research fraud. Conversely, preprinting servers (e.g., bioRxiv, medRxiv) that have recently emerged and constitute an alternative approach to assess the scientific integrity of a submitted manuscript quality are not as thorough as the peer review process. Therefore, manuscripts evaluated by preprinting servers that have not been peer-reviewed can often be invalid. Many social media sources use the results presented in preprinting servers to break sensational news, as in the case of COVID-19 in recent years (4, 5). To increase the effectiveness of identifying research fraud without overwhelming human efforts, many journals including the *Frontiers in Cardiovascular Medicine* have begun using artificial intelligence in the initial screening. Moreover, to guide the scientific community and investigators in this critical issue, the editors of *Frontiers in Cardiovascular Medicine* organized this Research Topic to discuss further the challenges in examining Research Reproducibility and Preventing Fraud.

Scientific rigor is the key term that many funding bodies and journals use to encourage multiple testing to eliminate biases that affect the interpretation of experimental or clinical data. Confirmation of the original results by other researchers is essential to validate the reported findings. This can be achieved only if there is a transparent description of the methods and a rigorous revision of the presented data. To what degrees such rigor and transparency are needed is a matter of question since strict guidelines have not been set yet. To this end, Moore et al. (Rigor Me This: What are the Basic Criteria for a Rigorous, Transparent, and Reproducible Scientific Study?) summarized the points to take into consideration when performing experiments and reporting experimental data for both *in vitro* and *in vivo* preclinical studies.

The science is built upon the previous findings by others to advance the field. It is therefore important to properly cite the published studies and discuss why the findings of the present study support or contradict the results of earlier analyses. Unfortunately, authors are often citing a paper that is irrelevant to the conducted research. Such errors might occur when many papers are cited in one manuscript as in the case of a review article. To prevent such unwanted or unintended outcomes, Li and Hung (How to reduce errors and improve transparency by using more precise citations) have provided recommendations on how to improve transparency and reduce the spread of incorrect information.

## Obligations of journals

Scientific journals are the media in which scientists communicate their findings to other scientists. In such media, peer review is important in keeping scientific integrity intact to prevent research fraud and misinterpretation of research results. Because of the publish-or-perish culture and the pressure from funding bodies, the speed and pressure to publish in scientific journals have intensified in recent years, resulting in the rise of so-called predatory journals to allow for a fast or superficial reviewing process to get accepted and published. This unfortunate byproduct of a flawed culture has caused numerous problems for scientists, universities, and funding bodies. As such, authors must be cautious and carefully consider the target journal they wish to choose to publish their research. To aid in understanding the trends in scientific journals, Cogan (Preventing fraud in biomedical research) summarized dos-and-don'ts in publication processes.

When one submits a manuscript to a journal, it is not always transparent how the editorial board will handle the manuscript. To guide authors submitting their manuscripts to *Arteriosclerosis, Thrombosis, and Vascular Biology*, an American Heart Association journal, Lu and Daugherty (Key factors for improving rigor and reproducibility: guidelines, peer reviews, and journal technical reviews) summarized their experience as editors to enhance rigor and reproducibility for preclinical research.

It is important to remember that the editors and reviewers provide their volunteer and unpaid hours while keeping scientific integrity. As the editors of *Frontiers in Cardiovascular Medicine*, we hope that authors would not intentionally forge the research data to get their manuscripts accepted in scientific journals and carefully consider their target journal to avoid publication in fraudulent journals with low-quality standards. Citing wrong publications to bend the scientific findings is an unacceptable practice that must be prevented. The articles published in this Research Topic will help guide researchers in improving research integrity and reproducibility and preventing fraud.

## Author contributions

SU drafted and revised the editorial. All authors edited, contributed to the article, and approved the submitted version.

## Conflict of interest

The authors declare that the research was conducted in the absence of any commercial or financial relationships that could be construed as a potential conflict of interest.

## Publisher's note

All claims expressed in this article are solely those of the authors and do not necessarily represent those of their affiliated organizations, or those of the publisher, the editors and the reviewers. Any product that may be evaluated in this article, or claim that may be made by its manufacturer, is not guaranteed or endorsed by the publisher.
